# Targeting Nucleotide Binding Domain of Multidrug Resistance-associated Protein-1 (MRP1) for the Reversal of Multi Drug Resistance in Cancer

**DOI:** 10.1038/s41598-018-30420-x

**Published:** 2018-08-10

**Authors:** Divya Dhasmana, Ashutosh Singh, Rohit Shukla, Timir Tripathi, Neha Garg

**Affiliations:** 1 School of Basic Science, Indian Institute of Technology Mandi, Kamand, Himachal Pradesh India; 2 Molecular and Structural Biophysics Laboratory, Department of Biochemistry, North Eastern Hill University, Shillong, India

## Abstract

Multidrug resistance (MDR) is the major cause, by which cancer cells expel the drugs out, developing a challenge against the current chemotherapeutic drugs regime. This mechanism is attributed to the over expression of ABC transporters like MRP1 on the surface of cells. Since nucleotide binding domains (NBD) of ABC transporters are the site of ATP binding and hydrolysis, thereby in this study we have targeted NBD1 of MRP1using molecular docking and molecular dynamic simulations (MDS). The compounds present in the FDA approved library were docked against NBD1 of the human multidrug resistance associated protein 1 (PDB ID: 2CBZ). For the docking studies, Standard Precision and Extra Precision methods were employed. After the EP docking studies, ligands showed an extremely low docking score that was indicative of very high binding affinity of the ligands to the NBD. Apart from the low docking score, another short listing criterion in simulation studies was the interaction of incoming ligand with the desired conserved residues of NDB involved in ATP binding and hydrolysis. Based on these measures, potassium citrate (DB09125) and technetium Tc-99m medronate (DB09138) were chosen and subjected to 100 ns simulation studies. From the MDS study we concluded that between these two compounds, potassium citrate is a better candidate for targeting MRP1.

## Introduction

The leading cause of chemotherapy failure is the multi drug resistance (MDR) exhibited by cancer cells due to which chemotherapeutic drugs are expelled out of the cells. The overexpressed efflux protein pumps belonging to ATP Binding Cassette (ABC) family^1^ are the key reason for MDR in cancer cells. The ABC superfamily can be divided into two major categories namely, ABC transporters which require ATP for substrate translocation and non-transporter proteins that are responsible for DNA maintenance and gene regulation^2^. In bacterial system, ABC transporters act as importers as well as exporters. They translocate molecules like ions, amino acids, sugar etc. inside and out of the bacterial cells. In contrary to this, only ABC family exporters are extant in a eukaryotic system. These drug pumps are an important part of a cell’s defense mechanism and this was first realized by DanØ who in 1973 demonstrated that Ehrlich ascites cells lower the concentration of intracellular daunorubicin by expelling it out^3^. After the discovery of P-glycoprotein by Ling & Juliano in 1976 as a 170 kDa transmembrane glycoprotein, a second type of drug transport protein i.e. multidrug resistance-associated protein (MRP) was later discovered by Chole in 1992^4^. It was cloned from H69R lung cancer cell line. Both the P-gp and MRP belong to the family of ABC transporters and both are responsible for conferring multidrug resistance to cells. In spite of a very low structural identity between MRP1 and P-gp, there was a considerable similarity between the phenotype of H69AR cells and cells expressing P-gp^5^.

MRP1, a 190 kDa protein is capable of exporting hydrophobic drug molecules, folates, glutathione conjugated compounds, glucornoid conjugated steroids and organic anions to name a few^6^. It is a 1531 amino acid protein with two Nucleotide Binding Domains (NBD) and three Membrane Spanning Domains (MSDs) namely MSD0, MSD1 and MSD2 consisting of 17 transmembrane helices (TM) deviating from the structure of a typical ABC transporter that consists of two MSDs consisting of 6 TMs^7^. The MSD0 is not essential for function but it becomes essential for trafficking protein in which the missing COOH- terminal is either mutated or missing thus explaining the different trafficking requirement of MRP1 as compared to other ABC transporter proteins^8^. Nevertheless, despite the numbers of MSDs present in an ABC transporter their NBDs share many conserved features, especially NBD1. The two NBDs play a crucial role in the transport activity of ABCC proteins.

One feature common to all ABC family members is the dependence of their ATPase activity on the cooperative interactions between the two NBDs. It has already been proved that both of the NBDs are involved in the transport of substrates across the cell and the binding and release of substrate depends on the ordered binding of ATP to the two NBDs. In addition to this, ATP binding is essential for the export of substrates converting the protein from a high-affinity to low affinity substrate binding state^9,10^. Moreover, ATP binding to NBD1 is not very much dependent on the functional state of NBD2 but for ATP binding to NBD2 is highly dependent on the functional state of NBD1^9,10^. The affinity of NBD1 for ATP is several times higher compared to NBD2^11^. Therefore, it can be possible that NBD2 merely acts as a site of ATP hydrolysis.

Molecular docking provides a platform where using the crystal structure of a protein as a template novel ligands can be determined. A library of compounds and a protein template is required to perform docking studies^12^. Docking has been applied mainly to enzymes and has been proved useful in finding the novel ligands with good substrate affinities. In order to discover a promising ligand for a macromolecule, it is important to understand the interactions and principles by which the ligand will recognize and interact with it. This forms the basis of structure based drug designing (SBDD). For SBDD, method of molecular docking is employed because it is able to predict the conformation and binding of a ligand into the active site of the macromolecule with preciseness. Using molecular docking, the binding mode of ligand and macromolecule, types of interactions (electrostatic, hydrophobic, pi cation) and the binding affinities can be analyzed^13^. Although docking algorithms have developed and improved over years, some issues like involvement of water molecules in the protein structure, conformational changes that occur following ligand binding still needs to be resolved^14^.

Molecular docking and MD simulations approach has already been used by many research groups to target MRP1 as well as P-gp. Williamson *et al*. used molecular docking analysis to predict the potential binding site of quercetin glucuronides on ABCC2 and then examined these predictions experimentally^15^. From this study it was concluded that molecular model agreed with the experimentally determined interactions between ABCC2 and ligand. In 2008, a study conducted on MRP5 demonstrated a model for this protein. Further potential binding sites were determined and with docking of guanosine 3′–5′ cyclic monophosphate (cGMP) the importance and involvement of transmembrane helix (TMH) was determined^16^. Sirisha *et al*. used molecular docking to find the inhibitors of MRP1 among dihydropyridine derivatives^17^. In addition to this, potential substrates for other ABCC members have been determined using molecular dynamics simulations. Furthermore, the inhibitory effect of curcumin on MRP1 has been studied by *in silico* studies.

Targeting ATP-binding site for drug discovery is not a new concept. The strategy has been explored against many proteins including tyrosine kinases. In 1999, Novartis Pharmaceuticals developed a pharmacophore model of the ATP-binding site present in Epidermal Growth Factor Receptor (EGFR)^18^. Since then this model has been used to find therapeutic molecules that can target the ATP binding site. In addition to this, with the help of docking and molecular dynamics studies on compounds like indirubins, it was predicted that these compounds bind at the active site where the adenine ring of the ATP binds^19^. Moreover, in order to target the ATP binding site of topoisomerase II a purine inhibitor scaffold was designed leading to identification of novel compounds^20^. In 2012, another research group targeted the ATP-binding site of type II topoisomerase by synthesizing 5-aryl-1, 3,4-thiadiazole coupled phthalimide derivatives^21^. These compounds were docked against the ATP-binding site of the protein. The compounds with best in silico results were further evaluated *in vitro*.

**Figure 1:**
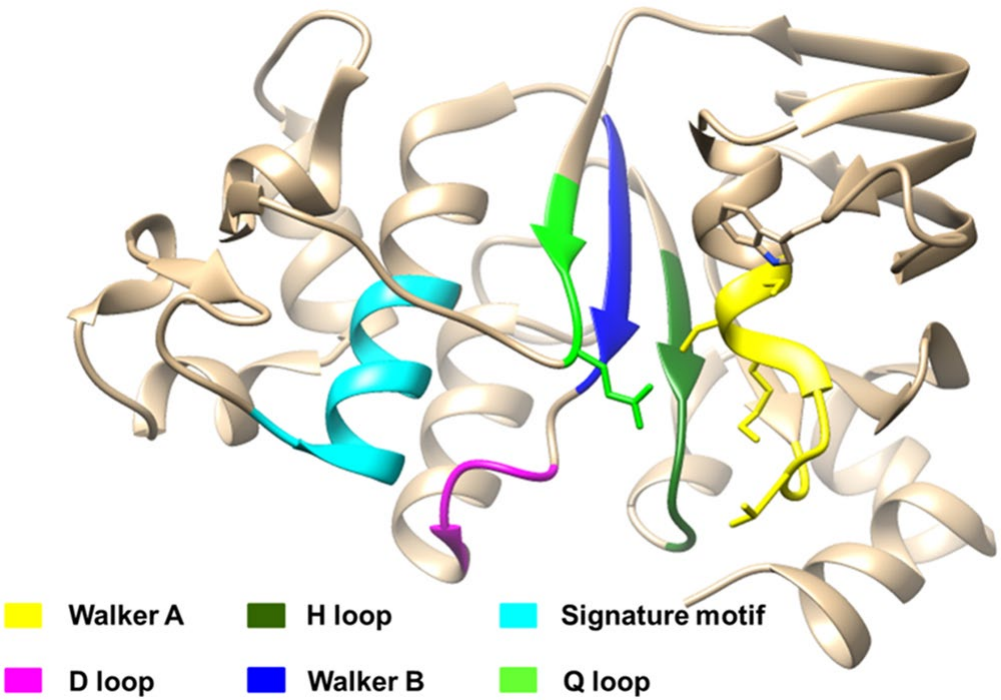
Ribbon diagram of the loops and motifs present in the NBD necessary for ATP binding and hydrolysis.

The ATP binding site for the docking studies was determined by previous studies. From the literature it was reviewed that NBD consists of some conserved motifs and loops that are essential for ATP binding and hydrolysis (Fig. 1). These include walker A which is a glycine rich motif responsible for ATP binding. Furthermore, walker B which consists of hydrophobic residues is also involved in ATP binding. The LSGGQ signature sequence also known as the C motif is placed opposite to the walker A and B of the other NBD and helps in completing the ATP binding sites^22,23^. The consensus D loop sequence (EATSALD) communicates with the other NBD^24^. In alpha-hemolysin translocation ATP binding protein HlyB, the H loop plays an essential role in ATP hydrolysis^25^ and in cystic fibrosis transmembrane conductance regulator it is required for the efficient closure of the channel^26^. Furthermore, the NBDs of the ABC family proteins share a high level of tertiary structure conservation. Therefore, an inhibitor that has been screened against the NBD of MRP1 can also be tried against the NBDs of other proteins from the same family. The transport cycle which involves the binding and consequently the hydrolysis of ATP is dependent on the walker A, walker B, D loop, H loop and Q loop of NBD. Walker A residues are responsible for H-bond formation between the α, β and γ phosphates and walker B motif is responsible for stabilization of ADP as well as for coordination of the magnesium ion. Residues in D and H loop (Asp792 and 793, and His827 respectively) are responsible for the stabilization of ATP. The Q loop has its role in the catalytic cycle in which the Gln713 residue makes contact with the magnesium ion and the attacking water molecule. Nucleotide binding is accompanied by structural changes in the protein involving walker A motif and the Q loop. Although the C signature sequence is not required by MRP1 for ATP binding, mutations in this sequence impedes the transition of protein from a high affinity binding state for substrate to a lower one. This can be attributed to the role of this sequence to form a correct interface for NBD dimer^27^. A similar phenomenon has been observed in case of P-gp^28,29^. Mutations in walker A and walker B of NBD2 did not affect the binding of ATP to NBD1 while on the other hand in case of NBD1, mutations in the Lys residue of walker can render the protein partially active or can eliminate the complete transport of leukotriene C4. Moreover, ATP is essential to change the high affinity substrate binding protein to a lower one. And thus it makes sense to target the NBD that is the site of ATP binding.

Within these conserved motifs and loops of NBDs, there are certain amino acid residues which are reported to be essential in ATP binding and hydrolysis. The conserved lysine residue located within the walker A is involved in ATP binding and when replaced by asparagine reduces the ATP hydrolysis activity in maltose/maltodextrin import (MaIK) as compared to the wild type^30^. The serine residue of the walker A forms H-bond with the Asp of Walker B and interacts with the Mg^2+^ and the β-phosphate of the bound ATP^31^. A conserved glutamate is present at the junction of walker B and D-loop is involved in co-ordination of magnesium ion^32^ and it also polarizes the incoming water molecules and acts as a catalytic base^33^. Mutation in this glutamate residue reduces the human Transporter Associated with Antigen Processing (TAP) protein transport activity as it is required for the abstraction of protons from the transition product formed^34^. However, this theory of glutamate acting as a catalytic base is disputed upon^25^. Study of the nucleotide binding domain of (TAP1) suggests that mutation in the D loop aspartate impedes the NBD dimerization^35^. In Q loop, glutamine residue is important as it is involved in co-ordination of catalytic metal and organizes the active site so that ATP hydrolysis can take place. The flexible nature of Q loop is utilized in separating the sub domains after the hydrolysis has occurred^36,37^. Additionally, the H loop histidine is involved in the transfer of protons in ATP hydrolysis^25^. Mutation in the conserved histidine of H loop reduces the ATPase activity in MaIK^30^. An interesting finding by Yang and research group suggests that a H-bond between H loop of NBD2 and ATP is essential for the ATP dependent transport of drugs by P-gp across the cell while mutations in H loop of NBD1 did not affect the protein conformation significantly^38^. Considering all this information about the NBDs of MRP1, it is likely to find an inhibitor that could make a complex with NBD1 thus hampering ATP binding.

The ATP switch and constant contact models are the two proposed models for ATP binding and hydrolysis in ABC transporters. In this model in the resting stage the two NBDs are wide apart without any ATP. As the ATP molecules start binding to the two NBDs sequentially, both the NBDs come together to form a dimer. After this one by one both the ATP molecules are hydrolyzed and converted to ADP and Pi. As the ADP and Pi are released, the protein reverts to the resting stage. On the other hand the constant contact model supports the alternating hydrolysis of ATP. In this the ATP-bound site is closed while the other one is empty and open ATP hydrolysis is followed by the binding of another ATP molecule and as a result of this binding the hydrolysis products of the first ATP bound are released. This cycle continues as long as ATP is available^2^.

**Figure 2:**
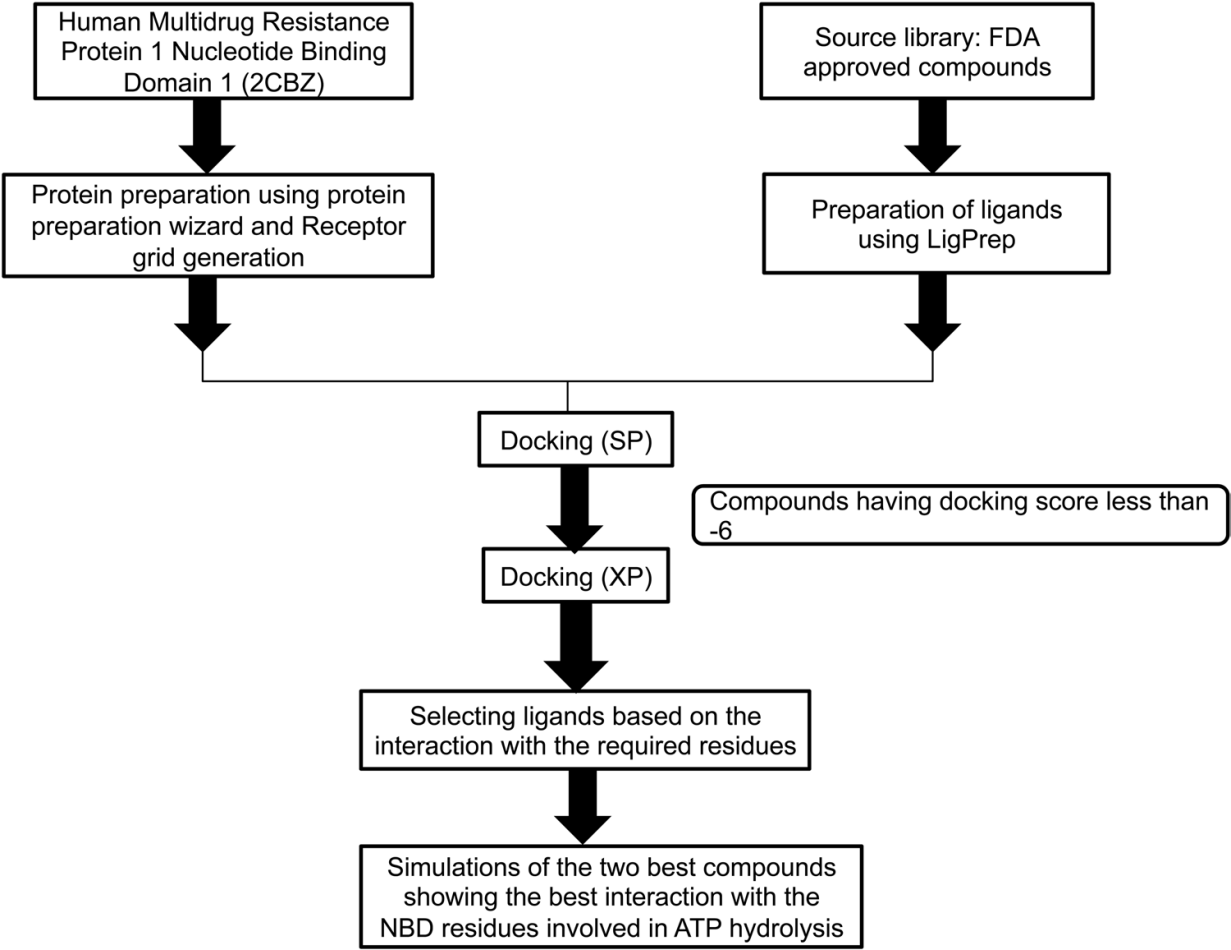
Flow chart depicting the methodology employed in the present study.

Based on this knowledge about the NBD domain of MRP1, the rational approach to screen for the inhibitors would be to check for the maximum number of interactions of the incoming ligand with the essential (target) residues of NBD. The target residues are those amino acids which are present in the loops and motifs of NBD and are essential for ATP binding and hydrolysis. In case of ABC transporters these residues are conserved. These include Gly681, Cys682, Lys684, Ser685 and Ser686 in Walker A motif, Glu713 in Q loop, Asp792 and Asp793 in D loop, and His827 in the H loop. The aim is to screen the molecules that will interact with maximum of these target residues as to obstruct ATP binding and hydrolysis. Considering this rationale, we have targeted the NBD 1 of the human multi drug resistance protein 1 to search for a compound that could halt the ATP binding and hydrolysis. For this, molecular docking studies were performed using FDA approved drugs as ligand to find the most suitable molecule. The FDA library was subjected to standard precision (SP) and extra precision (XP) mode of docking and the screened compounds demonstrated high binding affinity to the NBD domain that can be inferred by the low docking scores. Further the top hits were screened manually and the two prime ligands which were interacting with the maximum number of target residues of NBD were selected for molecular dynamic simulation studies. The flow chart for the methodology is shown in Fig. 2.

## Results

As per the information about the active site and the residues of the NBDs involved in ATP hydrolysis, it is rational to look for the interaction of these target residues with the ligand. The aim was to block these residues so that ATP would not get hydrolyzed and consequently, there would be no efflux of drugs. Eight residues (Gly681, Cys682, Lys684, Cys685, Ser686, Gln713, His827) were short-listed and then the interaction of ligands with the target residues was assessed using docking. On the basis of the docking score and the number of interactions of the ligand with the target residues, the compounds for MD simulations were shortlisted.

### Molecular docking studies

To assess the interactions between the incoming ligand and the active site of the protein docking was performed. First SP docking protocol was applied to shortlist the compounds. From the outcome of SP docking the compounds having lowest parameters were eliminated and a total of 68 compounds which were having a docking score less than −6 were chosen for XP docking. From the XP docking results (Supplementary Table S1), ligands having docking score less than −10 were selected and further manually screened for interactions with the target residues. Based on the highest binding affinity and the maximum number of interaction with the target residues (Supplementary Table S2), four compounds where selected from the XP docking result. Among the four compounds we identified that Potassium citrate (DB09125) has the highest binding affinity of −16.765 Kcal.mol^−1^ toward ATP binding site of NBD domain. The other three compounds i.e. Technetium Tc-99m medronate (DB09138), Polaprezinc (DB09221) and 4 Samarium (153 Sm) lexidronam (DB05273) showed the binding affinity of −13.059, −12.494 and −12.434 Kcal.mol^−1^respectively toward the active site (Supplementary Table S1).

### Analysis of the four best docked compounds

**Figure 3:**
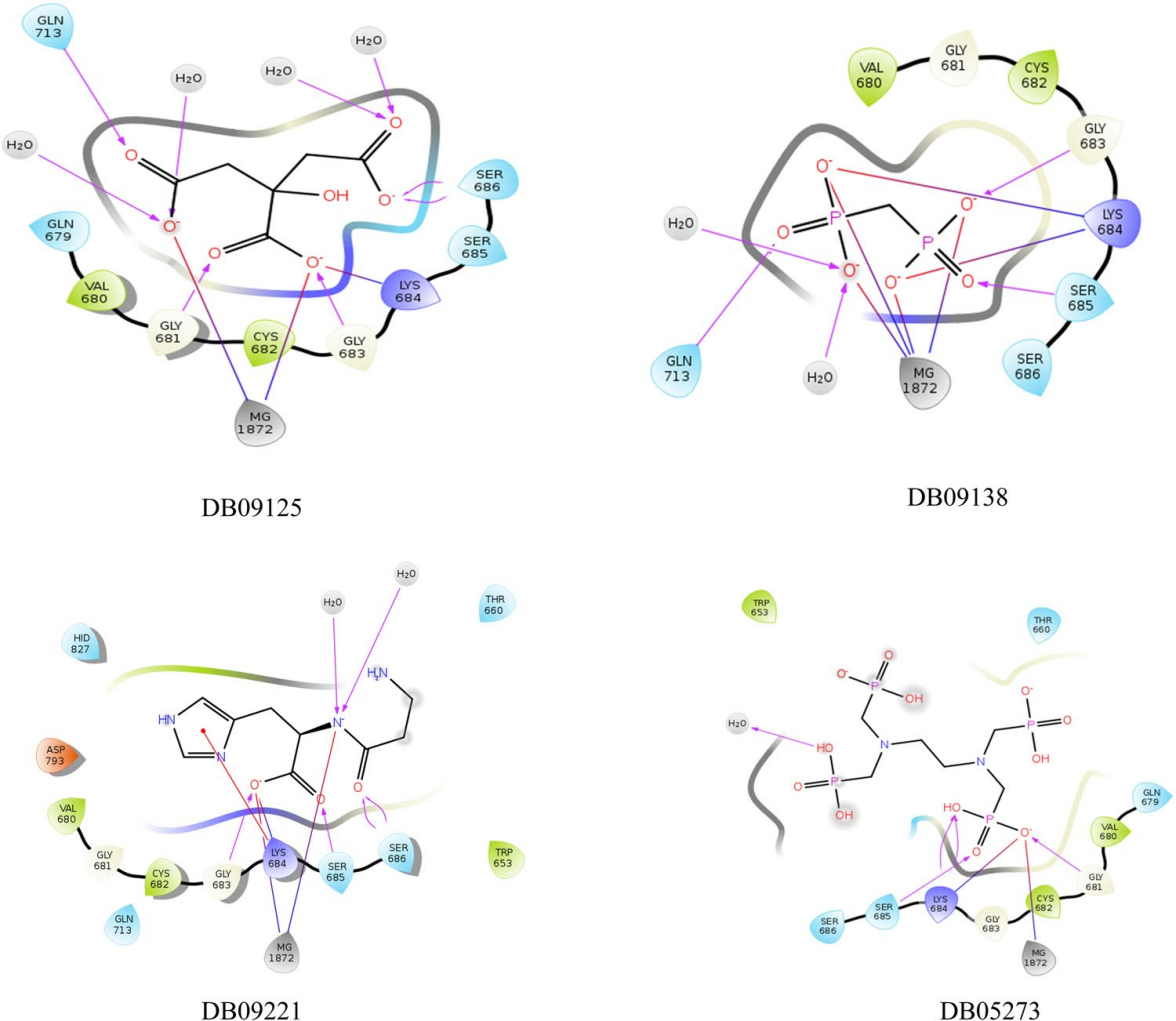
Figure showing 2D interaction diagram for the best hits i.e. DB09125 (top left), DB09138 (top right), DB9221 (bottom left) and DB05273 (bottom right).

**Figure 4:**
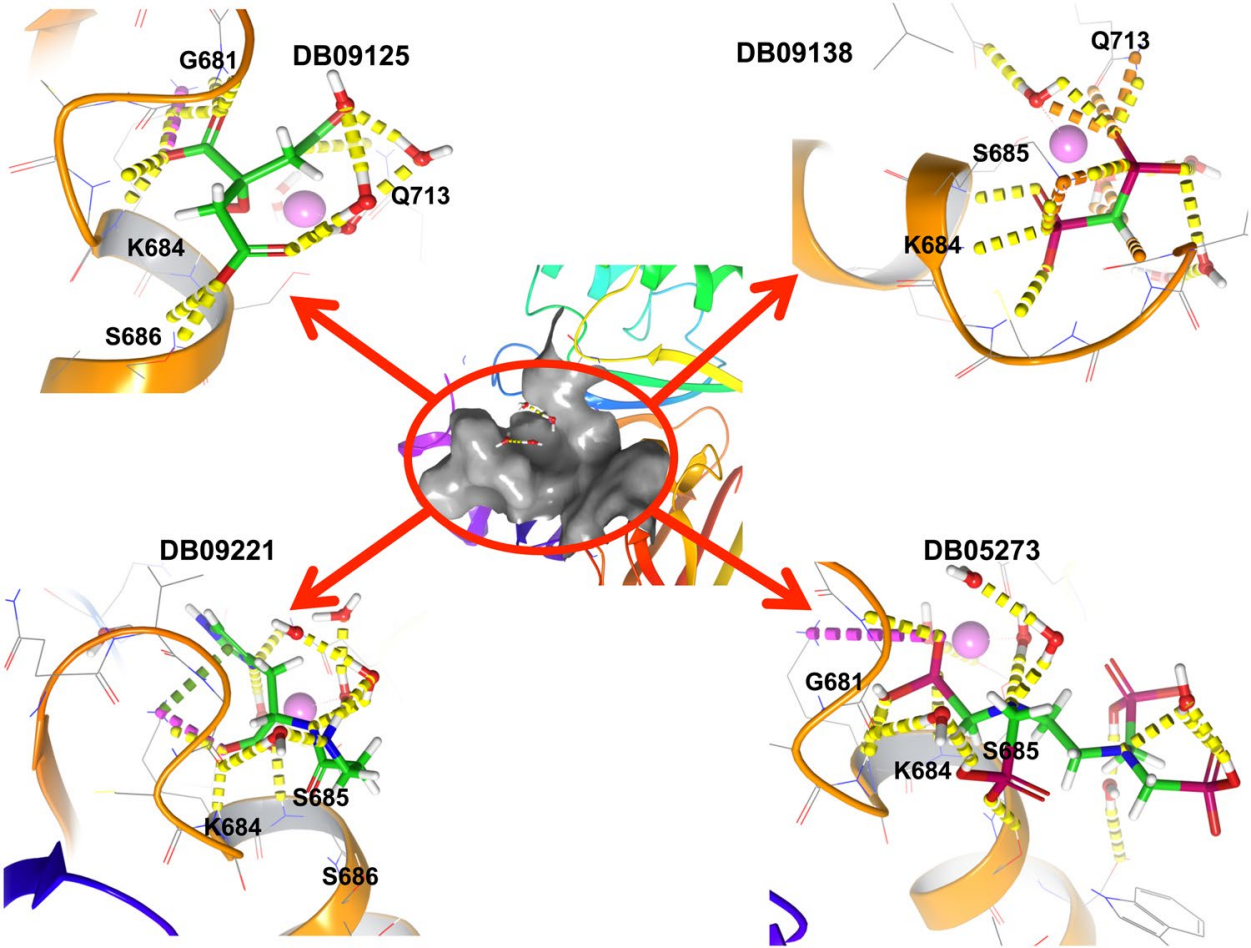
The potential binding poses for the best hits. The yellow dotted line represents intermolecular hydrogen bond interactions.

All the four top hit compounds selected on the basis of XP docking score and number of interaction with the target residues, showed a perfect fit inside the ATP binding grove (Figs 3 and 4) and interacted with the active site residues. The interacting residues, interacting target residues and residues in vicinity of the four compounds in the active site were explained individually.

### Potassium citrate (DB09125)

Potassium citrate showed the highest binding affinity toward ATP binding site of NBD domain with a docking score of −16.765. The compound interacted with four target amino acid residues with H-bonds (G681, S686, Q713) and salt bridge (K684). Additionally, one more H-bond interaction was observed with G683. Furthermore, the complex was stabilized by two salt bridge interactions with Mg^2+^ ion, a H-bond with surrounding water molecules and electrostatic interaction. Apart from this, other amino acid residues comprising the active site pocket consists of polar (Ser685, Gly679) and hydrophobic (Val680, Cys682) amino acids.

### Technetium Tc-99m medronate (DB09138)

Technetium Tc-99m medronate showed interactions with the target residues as well as with the other residues present in the active sites. The ligand also showed a high binding affinity towards NBD1 with a docking score of −13.059. It interacted with three target amino acid residues i.e. Ser685, Gln713 (withH-bond) and Lys684 (with two salt bridges). Further, it formed a H-bond with Gly683 and four salt bridges with Mg^2+^ in the active site. Other amino acids comprising the active site of NBD1 include hydrophobic (Val680, Cys682) and polar amino acids (Ser686, Gly681).

### Polaprezinc (DB09221)

Polaprezinc formed a salt bridge with Lys684, two H-bonds with Ser686, one H-bond with Ser 685 and one with Gly 683. Also it shared a pi cation interaction with Lys 684. Besides, it also formed two salt bridges with the Mg^2+^ion. The whole complex was stabilized by hydrophobic and electrostatic interactions. The other residues lining the active site include the polar amino acids His (827), Gln (713), Thr (660) and Gln (713), hydrophobic amino acids Val (680), Cys (682) and Trp (653) and a negatively charged Asp (793) residue.

### Samarium (153Sm) lexidronam (DB05273)

Samarium (153Sm) lexidronam formed an energy complex of −12.434 with the active site of NBD1. The compound interacted with three target residues and formed one H-bond with Gly681, two H-bonds and a salt bridge with Lys684 and one H-bond with Ser685 while a salt bridge was formed between the ligand and the Mg^2+^ ion. Overall, the complex was stabilized by polar interactions. Apart from the target residues the active site was composed of polar (Ser686, Gln679), non-polar (Gly686) and hydrophobic (Cys682, Val680)amino acid residues.

From the results of molecular docking (Figs 3 and 4), we chose two compounds for MD simulations. The two compounds were selected on the basis of two criteria; the docking score (Supplementary Table S1) and the number of interactions between the ligand and the target residues (Supplementary Table S2). Therefore, potassium citrate and technetium Tc-99m medronate were chosen. Potassium citrate demonstrated a docking score of −16.795 while it interacted with four target residues forming four H-bonds and a salt bridge. Technetium Tc-99m medronate formed an energy complex of −13.059 and displayed interaction with three target residues forming two H-bonds and two salt bridges.

### MDS

From the docking results the above two compounds were selected whichfulfilled both the criteria. The selected two complexes (potassium citrate and technetium Tc-99m medronate) with apo-NBD1 were employed for MDS study. MDS is a widely usable technique for understanding the dynamics of the system as well as stability of protein ligand complexes^39–42^. A total of three systems (apo-NBD1, NBD1-potassium citrate and NBD-technetium Tc-99m medronate) were created. After that all the systems were employed for 100 ns MDS study. All the systems got the equilibrated state after 40 ns hence last 60 ns stable trajectories were considered for further analysis. In this study, RMSD, RMSF, radius of gyration (Rg), H-bond, PCA, and binding free energy werecalculated.

### RMSD

**Figure 5:**
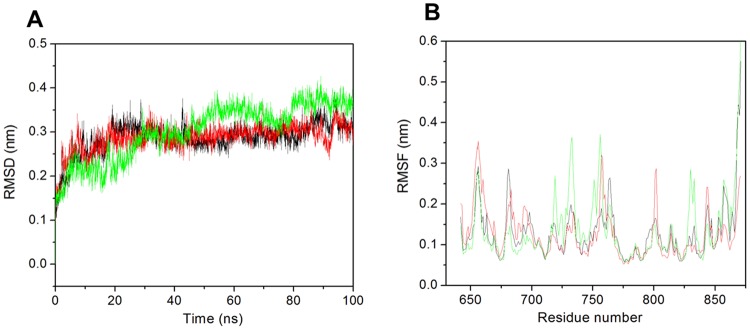
Molecular dynamics simulations. (**A**) RMSD of the Cα backbone of NBD1 (black) and the ligand complexes, NBD1-potassium citrate (red), NBD1-technetium Tc-99m medronate (cyan) over the 100 ns MDS at 300 K. (**B**) RMSF of residues during MDS for NBD1 (black) and the ligand complexes NBD1-potassium citrate (red) and NBD1-technetium Tc-99mmedronate (cyan).

The RMSD was used to measure the scalar distance between the atoms and for prediction of stability of the systems. The backbone RMSD indicated that all the systems are stable and well equilibrated after 40 ns and they produced stable trajectories for further analysis. The apo-NBD1 showed very stable RMSD average value of 0.28 while NBD1-potassium citrate and NBD1-technetium Tc-99m medronate complexes showed 0.28 and 0.30 nm RMSD respectively (Fig. 5A). The result indicated that NBD1-potassium citrate complex showed almost same RMSD as apo-NBD1 and formed much more stable complex as compared to NBD1-technetium Tc-99m medronate complex. From the RMSD result we concluded that all the systems are stable and NBD1-potassium citrate complex is more stable as compared to NBD1-technetium Tc-99m medronate complex.

### RMSF

The conformational changes during ligand binding were observed by the calculation of RMSF values. The RMSF describes the conformational changes of whole protein or each residue after ligand binding. The apo-NBD1 and both complexes (NBD1-potassium citrate and NBD1-technetium Tc-99m medronate) were used for RMSF calculation. The last 60 ns trajectories were considered for the calculation of RMSF. The average RMSF value for apo-NBD1, NBD1-potassium citrate and NBD1-technetium Tc-99m medronate complexes were 0.12, 0.12 and 0.13 nm respectively. It was observed that the NBD1-potassium citrate complex does not cause much fluctuation after binding and showed RMSF value like apo-NBD1 while the NBD1-technetium Tc-99m medronate complex showed higher fluctuation compared to this ligand. From the Fig. 5B we observed that apo-NBD1 and NBD1-technetium Tc-99m medronate complex showed higher fluctuation in the C-terminal domain while NBD1-potassium citrate does not show this fluctuation. From the RMSF result we concluded that NBD1-potassium citrate complex is novel as compared to NBD-technetium Tc-99m medronate complex.

### Radius of gyration

**Figure 6:**
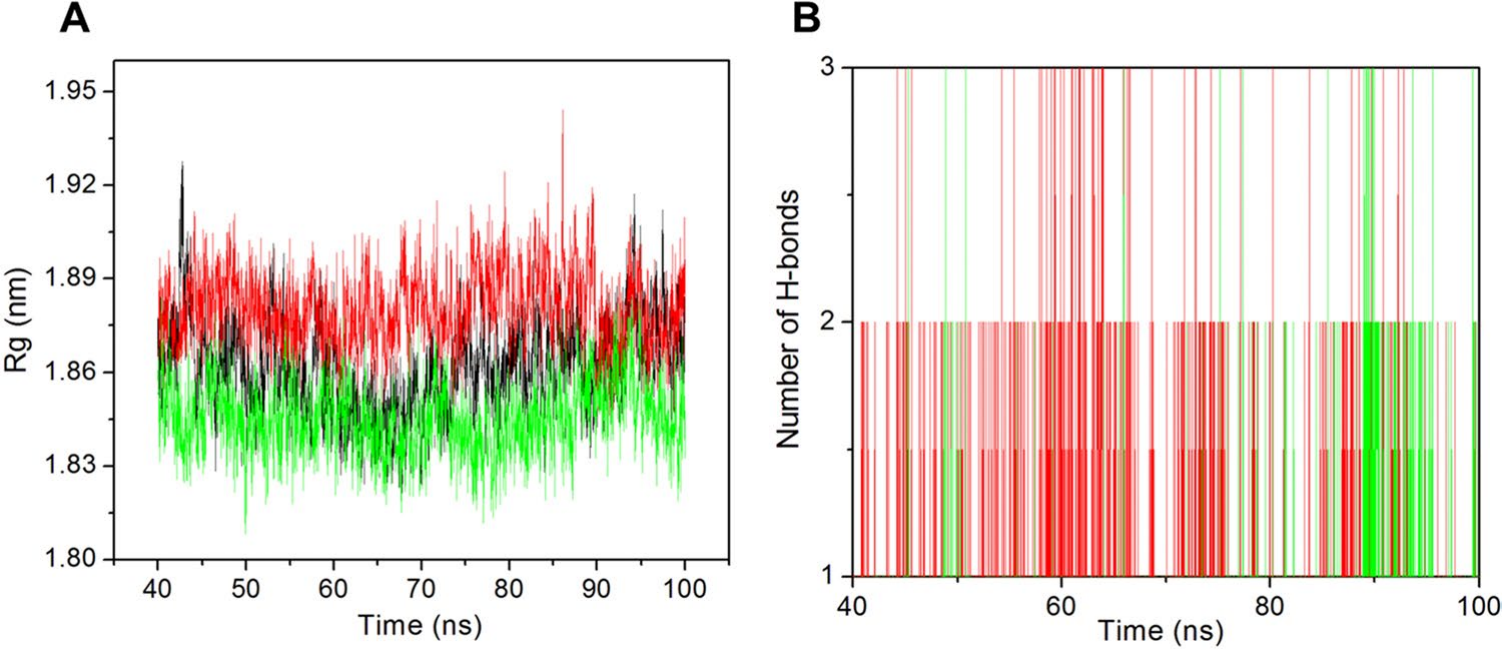
Structural stability analysis. (**A**) Plot of Radius of gyration vs time for NBD1 (black) and the ligands potassium citrate (red), NBD1-technetium Tc-99m medronate (cyan). (**B**) Number of hydrogen bond interactions formed between NBD1 and the ligands NBD1-potassium citrate (red), NBD1-technetium Tc-99m medronate (cyan) complexes during the last 60 ns simulation time period.

For prediction of the conformational changes and the compactness of the protein-ligand complexes, radius of gyration (Rg) was calculated for the last 60 ns (Fig. 6A). The Rg is defined as the mass weighted root mean square distance of a collection of atoms from their common center of mass. The average Rg value for apo-NBD1, NBD1-potassium citrate and NBD1-technetium Tc-99m medronate complexes were 1.86, 1.88 and 1.84 nm respectively. The Rg value clearly suggests that the NBD1-technetium Tc-99m medronate complex show least Rg value and forms a well compact complex than the NBD1-potassium citrate complex. It also show that after binding of the ligand, the protein attain a compact conformation in the case of NBD1-technetium Tc-99m medronate complex. The NDB1-2 complex showed higher Rg peak in the Fig. 6A. The result represents that both complexes are compact while NBD1-technetium Tc-99m medronate complex is more compact.

### H-bonds

The interaction between protein and ligand is transient and H-bonds play key role in the stability of protein-ligand complex. We have analyzed the number of H-bonds for last 60 ns trajectories (Fig. 6B). The average number of H-bonds for NBD1-potassium citrate and NBD1-technetium Tc-99m medronate complexes was 0–2 and 0–1 respectively. From the Figure we can clearly see that NBD1-potassium citrate complex show more number of H-bonds as compared to NBD1-technetium Tc-99m medronate complex. So the result clearly suggests that the NBD1-potassium citrate complex is more stable. To predict the key residues that play major role in H-bonding between the protein and ligand complex. The percent occupancy of H-bonds was calculated with the interacting residues from last 60 ns trajectory and shown in Supplementary Table S3.

### PCA

**Figure 7:**
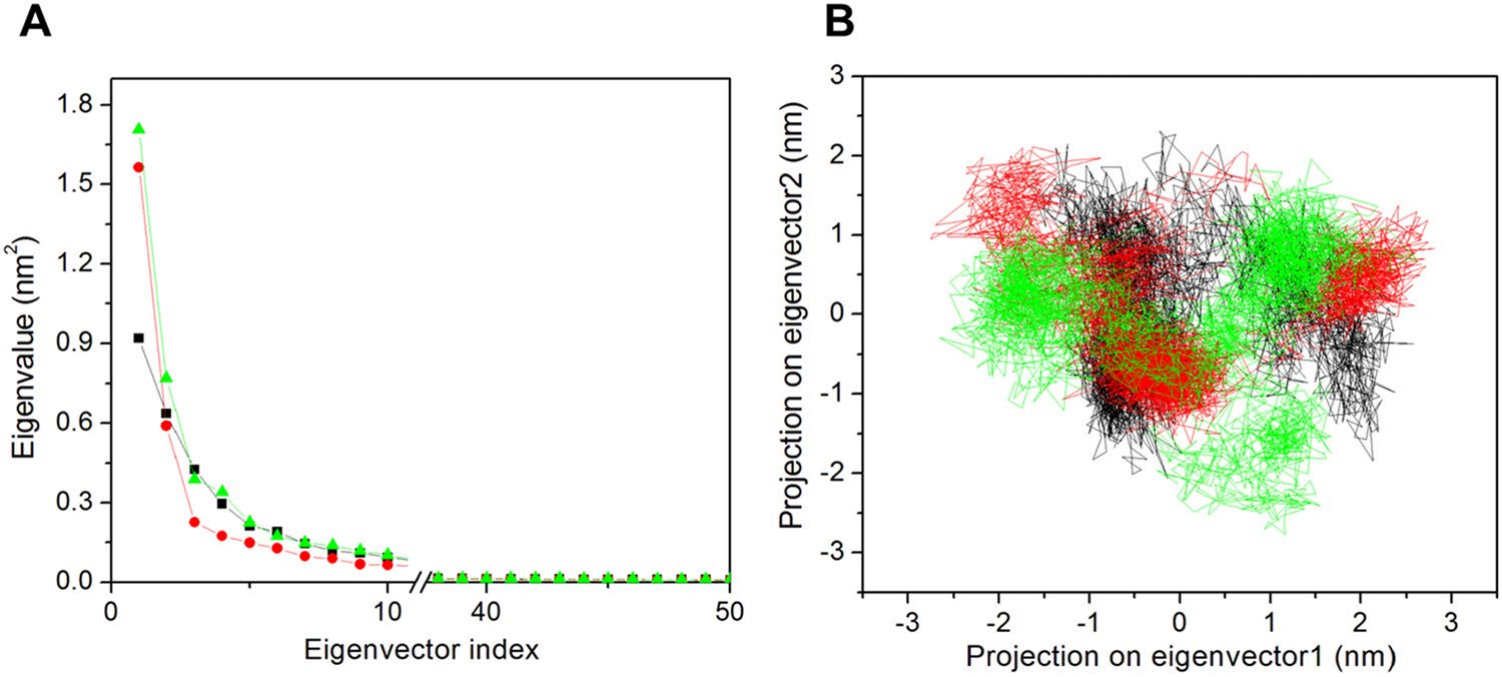
Principal component analysis. (**A**) Plot of eigenvalues was plotted vs eigenvector index. First 50 eigenvectors were considered. NBD1 (black) and the ligands, NBD1-potassium citrate (red), NBD1-technetium Tc-99m medronate (cyan) indicate the eigenvalues. **(B)** Projection of the motion of the protein in phase space along the PC1 and PC2 for NBD1 (black) and the ligands NBD1-potassium citrate (red), NBD1-technetium Tc-99m medronate (cyan) at 300 K.

The correlated motions during ligand binding were predicted by PCA. The first few eigenvectors play key role in the overall significant motions of protein. The first fifty eigenvectors were considered for the analysis. Covariance matrix of atomic fluctuations was diagonalized for predicting the eigenvalues. Figure 7A represents the eigenvalue in decreasing order versus corresponding eigenvector for apo-NBD1, NBD1-potassium citrate and NBD1-technetium Tc-99m medronate complexes. From this result we have analyzed ten eigenvectors that accounted for 74.76%, 78.89% and 79.45% motions for apo-NBD1, NBD1-POTASSIUM CITRATE and NBD1-technetium Tc-99m medronate complexes respectively. The correlated motions showed that apo-NBD1 showed lesser correlated motions as compared to the complexes. While in the case of only two complex the NBD1-technetium Tc-99m medronate showed higher motion and NBD1-POTASSIUM CITRATE showed lower motions. All these calculations were performed on the last 60 ns equilibrated trajectories. The results suggested that NBD1-potassium citrate complex could act as a potential inhibitor than NBD1-technetium Tc-99m medronate complex.

The 2D projection plot is a better way to clearly achieve the essential dynamics of the systems. We observed from the first 50 eigenvectors PCA results that the first two eigenvectors play crucial role in overall motions of the protein. So for predicting the conformational changes induced after ligand binding, we have drawn a 2D projection plot from the last 60 ns MDS trajectories (Fig. 7B). The figure clearly represents that apo-NBD1 showed less stable cluster as compared to both the complex but in the case of the two predicted ligand the NBD1-potassium citrate complex show more stable cluster than NBD1-technetium Tc-99m medronate complex. Thus we can conclude that NBD1-potassium citrate complex is more stable and that the 2D PCA result is in corroboration with the PCA result.

### Binding free energy analysis

The binding free energy was calculated for prediction of binding energy of the complexes using MM-PBSA tool. The results are shown in Supplementary Table S4. The last 10 ns trajectories were used for the calculation of binding free energy. The binding free energy also reveals that NBD1-potassium citrate complex has more favorable binding affinity than NBD1-technetium Tc-99m medronate complex. The NBD1-potassium citrate showed a binding energy of −85.31 kJ.mol^−1^ while NBD1-technetium Tc-99m medronate complex showed −51.53 kJ.mol^−1^ binding affinity. Furthermore, it was observed that the electrostatic interactions, non-polar solvation energy and Van der Waals interactions negatively complimented the overall interaction energy while the polar solvation energy had positively enriched the binding energy. So from the binding energy result we have concluded that NBD1-potassium citrate complex is more stable as compared to NBD2-12 complex.

## Discussion

From the results of molecular docking and MD simulations, we conclude that potassium citrate has a better binding affinity towards the active site of NBD 1 as compared to Tc-99m medronate and the NBD1- Potassium citrate complex was found to be more stable than NBD1-technetium Tc-99m medronate complex. From the docking analysis it was observed that the binding affinity of Potassium citrate (−16.795) was better compared to that of technetium Tc-99m medronate (−13.059). From the 2D ligand interaction diagram it was observed that potassium citrate interacted with maximum number of target residues (Gly681, Lys684, Ser686, Gln713) as compared to technetium Tc-99m medronate (Lys684, Ser685, Gln713). The walker A of NBD is responsible for stabilizing the incoming ATP by forming H-bond with its phosphates. Three of the four target residues of NBD1 that are showing interaction with the potassium citrate are conserved residues of walker A and are essential for ATP binding. All these three residues form H-bond with the phosphates of bound ATP^30,31^. Additionally, the fourth target residue that is showing interaction with potassium citrate is Gln of Q loop. This residue is crucial as it is involved in co-ordination of catalytic metal and organizes the active site so that ATP hydrolysis can take place^36,43^. The docking result shows that the ligand potassium citrate occupies the ATP binding domain of NBD and interacts with the crucial amino acid residues that are necessary for ATP binding and active site organization. Therefore, potassium citrate binding will obstruct the binding of ATP and subsequent hydrolysis. As already discussed in the introduction, ATP binding is essential to change the high affinity substrate binding protein to high affinity substrate binding protein. Thus, obstruction in ATP binding will lead to the inhibition of MRP1 and reversal of MDR in cancer cell. Moreover from the overall RMSD, RMSF, PCA, H-bonding, Rg and binding free energy results, it was shown that potassium citrate is a better ligand for the active site of NBD as compared to technetium Tc-99m medronate.

The high expression level of MRP1 leads to multidrug resistance in cancer cells. This poses significant problem as the chemotherapeutic drugs employed to kill cancer cells are expelled out. Therefore it becomes quite evident that MRP1 is an ideal target to tackle MDR in cancer cells. Compounds like verapamil have been used to tackle this problem but its use has been limited because of its cardiotoxicity and the search for inhibitors of drug pumps like P-gp and MRP1 is still going on. In the current scenario, researchers are exploring different approaches to target MRP1 including increased GSH flux mediated by MRP1 that increases the oxidative stress in cells, interactions of MRP1 with different compounds that could obstruct the substrate transport and targeting expression level of MRP1 at mRNA level^44^.ABC transporters work by binding and hydrolyzing ATP. As already discussed above many other ATPase proteins targeting their ATP binding domain is a well-known approach especially for tyrosinase kinases and topo isomerases. As the transport activity of ABC transporters is powered by ATP binding and hydrolysis targeting its NBD that contains the ATP binding site is worth trying.

With the help of molecular docking and MD simulations we have proposed potassium citrate, an FDA approved compound, for inhibiting the NBD of MRP1.Potassium citrate is not new to be used as a drug. It is an FDA approved compound that has been used in preventing reoccurrence of kidney stones. Additionally, it is also used to deter the age dependent bone density loss^45^. Researchers have shown that potassium citrate supplementation along with thiazide prevented stone formation in patients with hypocitraturia^46^. We have hereby proposed for the first time that potassium citrate can bind to the active site of NBD1. Therefore, potassium citrate can act as an inhibitor of MRP1 and thereby it has the potential to hamper the efflux of drug from the cancer cells. Although, further *in-vitro* studies are needed to ensure the inhibiting potential of potassium citrate. This present study only provides the docking and simulation confidence on potassium citrate to be the best candidate for targeting MRP1 among the FDA approved compounds library.

## Materials and Methods

### Protein preparation

The crystal structure of human Multi drug resistance protein 1 Nucleotide Binding Domain 1 (PDB ID: 2CBZ) with a resolution of 1.5 Å was taken from RCSB Protein Data Bank (PDB) (http://www.rcsb.org/pdb/). Protein was prepared using the “protein preparation” wizard of Schrödinger Maestro suite. The missing loops and missing side chains were filled and the water molecules, except for those present in the vicinity of the active site, were removed. In this way the protein structure was refined and finally optimized and minimized.

### Receptor grid generation

The prepared protein was used to generate the grid upon which the ligands were screened for their affinity towards the active site of protein. Receptor grid was generated using “Glide” module (Schrödinger), around the active site at which ATP along with Mg^2+^ was bound. Grid was generated in such a way that all the conserved motifs and loops involved in the binding and hydrolysis of ATP were included. The co-ordinates of the generated grid were x = −17.43, y = 47.21, z = 0.6.

### Ligand preparation

FDA approved compounds were used for ligand preparation (1996) and were prepared using “LigPrep” module (Schrödinger). LigPrep suite of Schrödinger can generate different states for a single ligand while looking for tautomers, isomers and ionization states. Using LigPrep, the ligands were optimized to possess lowest energy, right chirality and right ionization state. The pH was set to the physiological pH of 7.0. The ligands were optimized using OPLS-2005 force field. Once all the ligands were optimized, they were further considered for docking studies.

### Docking

To determine the interactions between the ligands and the active site, docking experiments were performed using the “Ligand Docking” module of Schrödinger. The prepared ligands were docked into receptor grid that was generated on the prepared protein. First the Standard Precision (SP) docking protocol was followed in which the ligands were sorted based on the docking score and glide score. Glide module of Schrödinger uses the Emodel scoring function to compare between the ligand-protein complex poses and the Glide score. It chooses the best pose and then compares the poses with the Glide score. From the result of SP docking, the compounds having lowest parameters were eliminated and then extra precision (XP) docking was performed.

### Molecular dynamics simulation (MDS)

MD simulations were performed in an *in-house* supercomputer using GROMACS 4.6.5^47,48^ with GROMOS 9653a6 force field and SPC216 water model as previously mentioned^49–52^. A total of three systems (one for apo-protein and two for protein-ligand complex) were created for 100 ns MDS. The ligand topology was generated by using PRODRUG server^53^. A cubic simulation box was created and filled with water molecules and then allowed for padding around the protein; Four Na^+^ ions were included to neutralize the system. Energy minimization was carried out using the steepest descent algorithm for removing the steric clashes of the systems. Then, all the systems were equilibrated under NVT and NPT for 1 ns for position restraint. The electrostatic interactions were computed with particle mesh Ewald (PME) method^54^. To compute Lennard-Jones and Coulomb interactions, 1.0 nm radius cut-off distance was used. The LINCS algorithm^55^ was used to constrain the H-bond lengths. The time step was maintained at 2 fs for the simulation. To predict the short-range non-bonded interaction, 10 Å cut-off distance was used. 1.6 Å Fourier grid spacing was used for the PME method for long-range electrostatics. All bonds including H-bonds were fixed by Shake algorithm^56^. The MD simulations were performed at 300 K temperature with the integration time step of 2 fs.

### Analysis of MD data

The analyses on the obtained trajectories were performed using GROMACS utilities. To understand the conformational changes, root mean square deviation (RMSD) and radius of gyration (Rg) of all backbone Cα atoms were computed with respect to the native structure at 300 K. The structural stability of the proteins was defined in terms of root mean square fluctuations (RMSF) and H-bond interaction to provide direct evidence of protein ligand stability. The RMSD and RMSF were calculated using g_rms and g_rmsf tools as mentioned earlier^52^ while the H-bonds were and principal component analysis (PCA) were carried out by using g_hbond, g_covar and g_anaeig tool of GROMACS package. The trajectories were analyzed using Chimera 1.10.2, and the graphs were plotted by Origin 6.0 software.

### Binding free energy calculation

The binding free energy of protein-ligand complexes was calculated using the molecular mechanics Poisson–Boltzmann surface area (MM-PBSA) method^54,57^. Free energy of solvation (polar + non-polar solvation energies) and molecular mechanics potential energy (electrostatic + Van der Waals interaction) were calculated by this tool. In this study, the last 10 ns of the MD trajectories were taken for the calculation of MM-PBSA.

## Conclusion

In this study we have described two compounds, potassium citrate and technetium Tc-99m medronate that showed the prospective of reversing MDR in cancer cells. These compounds were screened through rigorous molecular docking methods and subjected to simulations study. We conclude that NBD1-potassium citrate is more stable as compared to NBD1-technetium Tc-99m medronate and thus can be the lead compound against NBD1 of MRP1. Moreover, as the NBD domain is conserved among the members of ABC transporter family the ligand could prove its mettle against other drug pumps also. The experimental studies based on our findings would lead to a better understanding of working with potassium citrate to tackle MDR in cancer cells.

## Electronic supplementary material

Supplementary Information
