# Supplementary material for: Targeting Nucleotide Binding Domain of Multidrug Resistance-associated Protein-1 (MRP1) for the Reversal of Multi Drug Resistance in Cancer
Source: Sci Rep. 2018 Aug 10;8:11973. doi: 10.1038/s41598-018-30420-x (PMC6086895; doi:10.1038/s41598-018-30420-x)
Supplement: Supplementary file 1 — Supplementary Information [file 41598_2018_30420_MOESM1_ESM.pdf]

Targeting Nucleotide Binding Domain of Multidrug Resistance-associated Protein-1 (MRP1) for the Reversal of Multi Drug Resistance in Cancer

Divya Dhasmana[1#], Ashutosh Singh[1#], Rohit Shukla[2], Timir Tripathi[2], Neha Garg[1]*

[1] **School of Basic Science, Indian Institute of Technology Mandi, Himachal Pradesh, India;**

[2]**Molecular and Structural Biophysics Laboratory, Department of Biochemistry, North Eastern Hill University, Shillong, India**

[#]**Equal Contribution**

* **Corresponding Author:** N.G. (Email: neha@iitmandi.ac.in)

Supplementary Table S1. XP docking results of selected compounds.

Compound	Generic name	Docking Score (Kcal/mol)	Glide Score (Kcal/mol)
DB09125	Potassium Citrate	-16.795	-16.795
DB09154	Sodium Citrate	-16.795	-16.795
DB11093	Calcium Citrate	-16.795	-16.795
DB06784	Gallium citrate Ga 67	-16.481	-16.481
DB09162	Ferric Citrate	-16.481	-16.481
DB09147	Ferric pyrophosphate	-15.892	-15.892
DB09165	Technetium Tc-99m pyrophosphate	-15.784	-15.784
DB00326	Calcium Gluceptate	-15.175	-15.175
DB09275	Bismuth Subcitrate	-13.597	-13.597
DB11126	Calcium gluconate	-13.069	-13.069
DB09138	Technetium Tc-99m medronate	-13.059	-13.059
DB09561	Sodium glycerophosphate	-12.723	-12.723
DB09425	Indium In-111 pentetate	-12.652	-12.652
DB09221	Polaprezinc	-12.494	-12.494
DB05273	Samarium (153Sm) lexidronam	-12.434	-12.434
DB00789	Gadopentetatedimeglumine	-12.095	-12.095

Supplementary Table S2. Table showing details of compounds that demonstrated maximum number of interactions with the target residues.

Compound ID	Generic name	Total no. of interactions	Target residues interacting with the ligand
DB09125	Potassium citrate	H-bonds: Gly683,Gly681, Ser686, Gln713<br>salt bridge: Lys684	Gly681, Lys684, Ser686, Gln713
DB09138	Technetium Tc-99m medronate	H-bonds: Ser685, Gln713<br>Salt bridges(2):Lys684	Lys684, Ser685, Gln713
DB09221	Polaprezinc	4 H-bonds: Gly683, Ser685, Ser686 (2)<br>salt bridge: Lys684<br>pi-cation: Lys684	Lys684, Ser685, Ser686
DB05273	Samarium (153Sm) lexidronam	H-bonds: Gly681, Lys684(2), Ser685<br>salt bridge: Lys684	Gly681, Lys684, Ser685

Supplementary Table S3. Table showing percentage occupancy of hydrogen bonds between protein and ligand.

Donor	Acceptor	Occupancy
**NBD1-POTASSIUM CITRATE**		
LIG2-872-Side	CYS682-Main	1.40%
SER686-Side	LIG2-872-Side	0.17%
CYS682-Main	LIG2-872-Side	0.17%
LIG2-872-Side	SER686-Side	0.17%
LIG2-872-Side	GLY843-Main	0.27%
LIG2-872-Side	THR660-Main	0.07%
VAL680-Main	LIG2-872-Side	0.73%
GLY844-Main	LIG2-872-Side	0.03%
GLY843-Main	LIG2-872-Side	0.67%
LIG2-872-Side	MET841-Side	0.17%
LIG2-872-Side	VAL676-Main	0.33%
GLY683-Main	LIG2-872-Side	0.20%
CYS682-Side	LIG2-872-Side	0.03%
LIG2-872-Side	GLY683-Main	0.03%
LEU687-Main	LIG2-872-Side	0.07%
**NBD1-technetium Tc-99m medronate**		
CYS682-Side	LIG12--Side	0.60%
THR660-Side	LIG12--Side	0.13%
LIG12--Side	THR660-Main	0.17%
TRP653-Side	LIG12--Side	0.20%
LIG12--Side	PRO659-Main	0.03%
LIG12--Side	SER685-Side	0.07%
LIG12--Side	GLY843-Main	0.03%
LIG12--Side	CYS682-Side	0.03%
LIG12--Side	SER689-Side	0.07%
ASN662-Main	LIG12--Side	0.17%
SER686-Side	LIG12--Side	0.03%
GLY844-Main	LIG12--Side	0.03%

MET849-Main	LIG12--Side	0.03%
ILE846-Main	LIG12--Side	0.03%
THR665-Main	LIG12--Side	0.37%
LIG12--Side	THR665-Main	0.10%
LIG12--Side	SER847-Main	0.03%
SER667-Main	LIG12—Side	0.37%
LIG12--Side	SER667-Main	0.03%

Supplementary Table S4. Table showing the Van der Waals, electrostatic, polar salvation, SASA and binding energy in kJ mol^{-1} for NBD1-POTASSIUM CITRATE and NBD1-technetium Tc-99m medronate complexes.

S. No.	Compound	Van der Waals energy	Electrostatic energy	Polar salvation energy	SASA energy	Binding energy
1.	NBD1-potassium citrate	-130.64 ± 9.08	18.74 ± 6.62	74.63 ± 8.54	-10.56 ± 0.55	-85.31 ± 10.37
2.	NBD1-technetium Tc-99m medronate	-68.41 ± 10.31	-2.88 ± 8.22	28.37 ± 9.08	-8.61 ± 1.18	-51.53 ± 10.02
